# Advances in Lung Cancer Imaging and Therapy

**DOI:** 10.3390/cancers14010058

**Published:** 2021-12-23

**Authors:** Egesta Lopci, Silvia Morbelli

**Affiliations:** 1 Nuclear Medicine, IRCCS Humanitas Research Center, Via Manzoni 56, 20089 Rozzano, Italy; 2 IRCCS Ospedale Policlinico San Martino, Nuclear Medicine, Largo Rosanna Benzi 10, 16132 Genoa, Italy; silviadaniela.morbelli@hsanmartino.it; 3 Department of Health Sciences (DISSAL), University of Genoa, Via Antonio Pastore 1, 16132 Genoa, Italy

**Keywords:** lung cancer, NSCLC, immunotherapy, combination therapy, radiotherapy, molecular imaging, radiomics, response assessment

This series of eight papers (five original articles, two reviews and one meta-analysis) is presented by international leaders covering various aspects of lung cancer management, starting with diagnostic imaging and analyzing the novel perspectives of therapy.

The target of the Special Issue was to provide an overview of the validated imaging techniques and future biomarkers for predicting responses to treatment, with particular focus on immunomodulatory regimens. In this regard, three original articles were directly dedicated to the use of metabolic information with [^18^F]FDG PET/CT singularly or in association with immune metabolic scores in the context of non-small cell lung cancer (NSCLC) treatment with checkpoint inhibitors [1,2,3]. In the first paper from Bauckneht and colleagues [1], 45 patients showing radiological progression according to RECIST 1.1 criteria during nivolumab administration have been evaluated. The authors investigate the role of the immuno-metabolic prognostic index [4,5] obtained as a combination of metabolic tumor volume (MTV) and systemic inflammation indexes (SII) to assess patient outcomes. Given the independent predictive value of MTV and SII with respect to patients’ overall survival (OS), the authors showed that their combination in the immune metabolic prognostic index (IMPI) could help to identify patients who would benefit from immunotherapy continuation, despite radiological progression [1]. In line with the aims of the issue also are the results of the paper published by Seban and colleagues on NSCLC patients undergoing first line immunotherapy with pembrolizumab [2]. Therein, the derived neutrophils-to-lymphocytes ratio (dNLR) had been combined with the total metabolic tumor volume (TMTV) on baseline [^18^F]FDG PET/CT, classifying patients into risk groups (poor, intermediate and low) to predict patient outcomes [6]. As expected, the poor prognosis group showed a significantly worse disease control rate (DCR) and overall response rate (ORR), as well as shorter progression-free survival (PFS) and OS. The proven prognostic impact of volumetric parameters on baseline [^18^F]FDG PET/CT has prompted also the report of Monaco and colleagues [3], who documented the real-life experience of the significantly longer OS and the better disease control rate of NSCLC patients treated with various regimens relating to checkpoint inhibitors (i.e., nivolumab, pembrolizumab or atezolizumab) and who were presenting with lower values of MTV and total lesion glycolysis (TLG) before starting treatment.

The exponential number of studies published in the last decade on immunotherapy response monitoring [7] demanded a special insight on the use of blinded independent central reviews (BICRs) in clinical trials management [8], as well as the careful monitoring of novel therapeutic drugs in lung cancer [9]. Both aspects were properly covered in the present issue by dedicated review articles. Beaumont and colleagues [8] firstly analyzed six lung cancer BICR trials that included 1833 patients overall. Based on this review, intelligent reading system implementation along with appropriate reader training and monitoring seem to mitigate most of the commonly encountered reading errors. The relevance of adequate central reviewing is enhanced in the case of new drugs development. As shown by the paper from Stencel and colleagues [9], new targets in NSCLC have been explored, including NTRK, MET, RET and HER 2 genes, with some of the particles having already received FDA approval. We cannot forget the frontier of combined treatments with different modalities, such as in case of radiotherapy associated with checkpoint inhibitors. This was in fact the focus of the article herein presented by Fiorica and colleagues [10], who aimed to report the magnitude of its benefits and potential clinical predictors. In their meta-analysis of 8435 patients with advanced or metastatic NSCLC, the combination of immune checkpoint inhibitors (ICI) with radiation therapy (ICI-RT) proved to increase OS by 1 to 3 years and progression-free survival compared to ICI or RT alone. Given the potential toxicities associated with RT addition, the significant benefit becomes a major incentive for clinicians. Hence, the evaluation of low-dose irradiation toxicity on the heart and lungs after thoracic radiotherapy has been the target of the paper from Schröder and colleagues [11]. Last but not least, the implementation of the apparent diffusion coefficient (ADC) histogram analysis for discriminating lung cancer from other inflammatory or infectious processes, such as pulmonary abscess and mycobacterial infection, was the focus in the cohort of patients analyzed by Usuda and colleagues [12].

Lung cancer is the most common cause of cancer-related death worldwide, and efforts to further improve the prognosis for advanced NSCLC are still ongoing. In this framework, the treatment algorithm for lung cancer has experienced a rapid evolution in the last few years and some of the newer therapeutic options are associated with increased survival. As a result of the availability of new therapeutic options, recent years have also witnessed great advances in the subfield that concerns the imaging of metastatic or advanced NSCLC. Emerging data are showing how we can improve the accuracy of disease relapse identification and broaden patients’ selection for new treatments, as well as helping us refine and standardize our response to therapy assessment, even in the case of combined therapies. Several of these topics are covered in the present Special Issue, showing the added value of multi-modal imaging (i.e., diffusion MRI imaging, PET) in different clinical settings. The next step will be the effective transfer of all these advances to clinical practice. For a final transfer of new imaging tools and analytic approaches to clinical practice, it will be necessary to incorporate imaging (and non-imaging) biomarkers in a single pipeline to guide advanced NSCLC patients risk stratifications before and after treatment.
